# Canine *ex vivo* tarsal arthrodesis: fixation by using a new bone tissue glue

**DOI:** 10.3389/fvets.2023.1250147

**Published:** 2023-09-20

**Authors:** Tobias Per Otto Lundin, Michael Pujari-Palmer, Gustaf Svensson, Odd Viking Höglund

**Affiliations:** ^1^Department of Surgery, Blå Stjärnans Djursjukhus, Gothenburg, Sweden; ^2^Department of Clinical Sciences, Swedish University of Agricultural Sciences, Uppsala, Sweden

**Keywords:** arthrodesis, fixation, stability, biomechanical model, glue, adhesive, resorbable

## Abstract

**Introduction:**

Arthrodesis, performed as a salvage surgical procedure to treat intractable joint conditions in dogs and cats, is associated with a high incidence of complications intra and postoperative, proving the need for improved and new techniques in arthrodesis surgery. Adding a new resorbable bone glue to the arthrodesis could potentially add fixation strength and lower complications. The objectives of this experimental *ex vivo* biomechanical study were therefore to develop a biomechanical test model of partial tarsal arthrodesis and to determine whether the new resorbable bone glue (phosphoserine modified cement) produced measurable fixation strength in canine calcaneoquartal arthrodesis, without orthopedic implants.

**Methods:**

Four biomechanical test models with a total of 35 canine tarsal joints were used. Soft tissues were dissected to 4 different test models with variable contributions from soft tissues. The calcaneoquartal joint was prepared as *in vivo* arthrodesis and the glue was applied to joint surfaces as a liquid/putty (0.4 cc). After curing for 24 h, a shear force was applied to the joint (1 mm per minute) and the failure strength was recorded.

**Results:**

Calcaneoquartal joints, where all soft tissues had been completely resected and fixated with glue (1–1.5 cm^2^ joint surface), withstood 2–5 mm of displacement and an average of 100 ± 58 N/cm^2^ of shear force (Model 1). Similar adhesive fixation strengths were obtained in Model 2 and 3 with increasing contributions from soft tissues (80 ± 44 and 63 ± 23 N/cm^2^, *p* = 0.39, ANOVA).

**Conclusion:**

The developed biomechanical model was sensitive enough to measure differences in fixation strengths between different glue formulations. The average fixation strength (60–100 N/cm^2^) should be strong enough to support short-term load bearing in medium sized canines (20 kg). The developed cadaver biomechanical test model is of potential use for other arthrodesis studies. The new resorbable glue can potentially contribute to stability at arthrodesis surgery, acting as a complement to today’s standard fixation, metal implants.

## 1. Introduction

Arthrodesis of the tarsal joint is a delicate surgery performed as a salvage surgical procedure, used in dogs and cats to treat intractable tarsal conditions. Examples are fractures, ligament ruptures, shearing injuries, osteochondral diseases, infections, malformations, and injuries to the Achilles mechanism (1–6). Arthrodesis is associated with a high incidence of complications (up to 80%) ranging from minor complications like cast complications, difficult wound closure and dehiscence, limb swelling and gastrocnemius tendinopathy, to major complications like plantar necrosis, infections, implant failure and misplacement, varus/valgus/rotational malunion and delayed or non-union of the arthrodesis (6–9). Pantarsal arthrodesis, fusion of the talocrural, intertarsal and tarsometatarsal joints, have higher incidences of major complications than partial tarsal arthrodesis (ParTA), fusion of the intertarsal and/or tarsometatarsal joints (6).

The high incidence of postoperative complications and intraoperative difficulties, show the need for new and improved techniques that may enable lower complication rates in arthrodesis surgery. An adhesive that facilitates implant placement and joint alignment, and adds strength to the internal fixation, could potentially be a useful complementary clinical tool to reduce the risk of complications. Furthermore, cases where postoperative external coaptation still are deemed necessary may potentially be reduced (2, 6–8, 10, 11).

In this experimental *ex vivo* study a new bone glue was evaluated as a subchondral bone adhesive (12–16). The glue is composed of natural materials that are present in the body, calcium, silicate, and the amino acid phosphoserine. Prior testing *ex vivo* and *in vivo*, has shown that the glue bonds to cortical bone, with an average bond strength of 100–400 N cm^−2^ (14, 16). The glue is resorbable *in vivo* when implanted into cancellous bone, and maintains bonding and fixation strength as it is actively reabsorbed by the body and turned into new bone, without excessive or pathological inflammation (14). The components of the glue can be sterilized by gamma irradiation and will react and function comparably. Depending on the formulation mix, the glue can be tailored to quicker solidification/hardening (30 s) or slower (10 min), with full strength after 2–4 h or 12–24 h, respectively, and with identical bond strength (Data on file). We hypothesized that adding the glue to an arthrodesis of the calcaneoquartal joint would increase the stability intra and postoperatively. This could potentially facilitate alignment, implant placement and reduce complications post operatively.

No similar, non-synthetic, degradable subchondral bone glue is approved for clinical use. Furthermore, there is no already-existing biomechanical test model, where a glue is applied directly at the arthrodesis joint, which can be used to evaluate an adhesive force. The first objective of this study was therefore to develop a cadaver biomechanical test model of canine ParTa, aimed at detecting loading forces during failure of a glue. Our second objective was to use the biomechanical model to determine whether the glue produced measurable, fixation strength (24 h) in an arthrodesis of the calcaneoquartal joint, without additional orthopedic fixation.

## 2. Methods

### 2.1. Materials

Canine cadaver hind limbs (left and right), from dogs euthanized for reasons unrelated to this study, showing no macroscopic signs of musculoskeletal diseases, were used. The cadavers were donated by owners via a signed consent form (at the time for the dog’s euthanasia). Ethical approval for use of cadavers was obtained from the Uppsala Animal Ethics Committee, 15533-2018, 04682-2020.

The adhesive was prepared from calcium silicate (AB Sigma-Aldrich Sweden, Stockholm, Sweden) and phosphoserine (Flamma SpA, Chignolo D’isola BG, Italy), and mixed with deionized water.

### 2.2. Sample preparation

Canine hind limbs were stored at −20°C wrapped in plastic bags and thawed (4°C) for 48 h prior to use. On the day of testing the limbs were kept in room temperature until they reached ambient temperature (21–22°C). The limbs were disarticulated at the talocrural joints. The skin was dissected from the calcaneoquartal and tarsometatarsal 4–5 joints. Tarsometatarsal joint 4–5 were completely disarticulated to prevent interference with movement in the calcaneoquartal joint. In arthrodesis of the calcaneoquartal joint *in vivo*, depending on type of injury, varying degrees of soft tissues are intact. To imitate this, the joints were randomly divided into 4 models where soft tissues were partially, or completely dissected.

**Figure 1:**
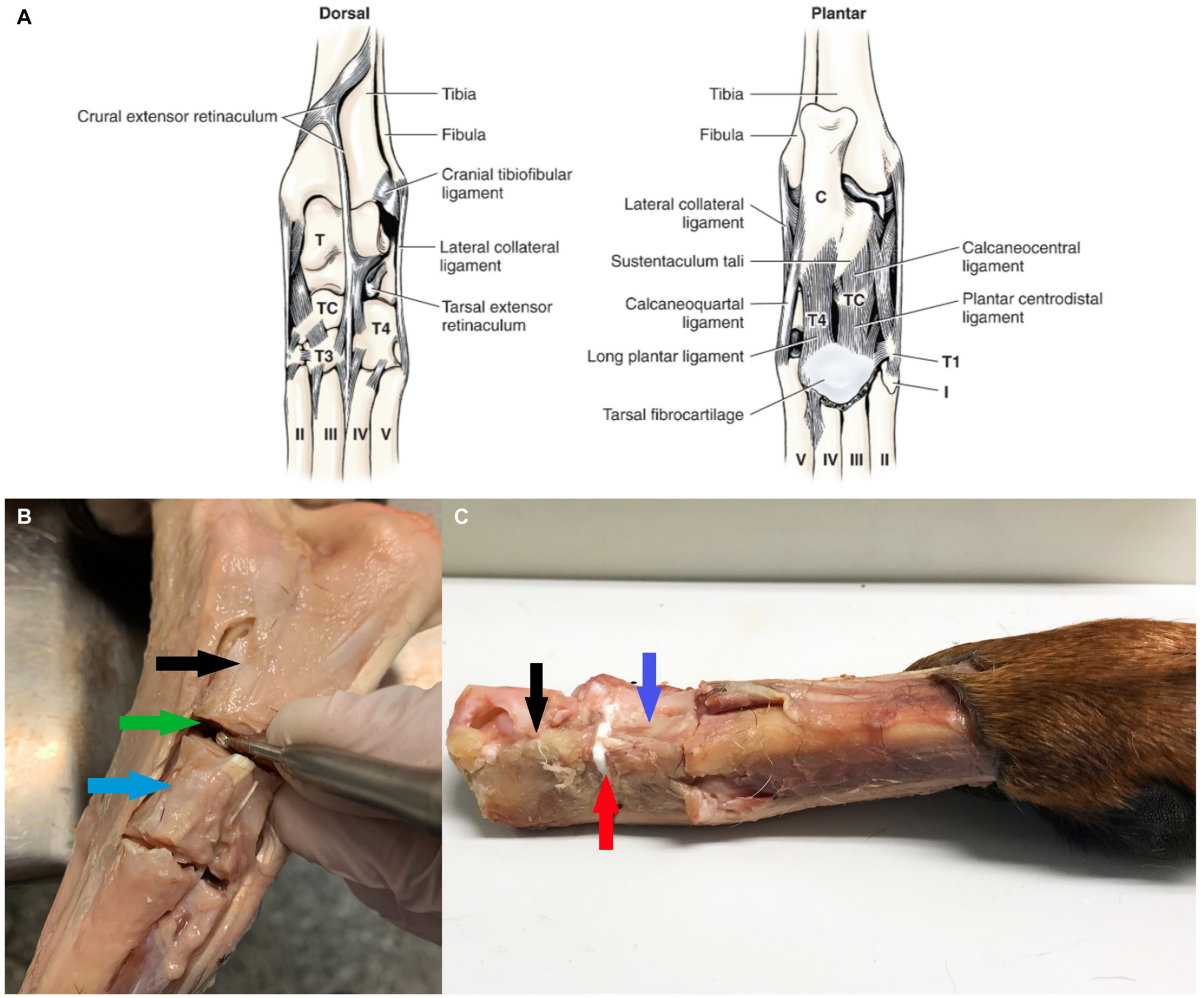
Overview of tarsal anatomy and tissue preparation. **(A)** Ligaments of left tarsus (From Evans and de Lahunta (17), with permission). **(B)** The soft tissues surrounding the calcaneal-quartal joint was resected, the joint debrided of cartilage. Black arrow highlights the calcaneus, blue arrow quartal bone and green arrow the calcaneal-quartal joint. **(C)** Glue was applied to the calcaneal-quartal joint, white colored adhesive is visible as bond line, highlighted by red arrow.

In Model 1 the proximal intertarsal joints were completely disarticulated. Model 1 represents a “purely shear” test, without contributions from any soft tissues. In Model 2 the lateral collateral, calcaneoquartal, long plantar and calcaneocentral ligaments, tarsal extensor retinaculum and flexor tendons were cut with the joint capsule of the calcaneoquartal joint. In Model 3 the lateral collateral ligament was cut off the calcaneoquartal joint (Figure 1A).

Since both proximal and distal ends were fixated in a test rig for Model 2 and Model 3, the trochlea of the talus was cut off with a band saw to create a level surface, for gripping. For comparison between the biomechanics of intact joints and our models 1–3, a completely intact tarsal joint (no resection of any soft tissue) was tested (Model 4). Then the calcaneoquartal joint was exposed, only by a minimal dorsal approach preserving all medial, lateral and plantar ligaments (Model 4-Glue), to determine whether the presence of intact soft tissues masked the effects of the glue (e.g., load sharing). In all models a high-speed 2–3 mm burr (Medtronic Integrated Power Control EC 300, Medtronic, Fort Worth, Texas), depending on the size of the dog, was used to meticulously remove cartilage from the calcaneoquartal joint surfaces (Figure 1B). Cartilage and bone remnants were flushed away and the diameter of the calcaneus and quartal bone surfaces where measured, tissues were then stored at −20°C until use.

The adhesive was prepared from pre-mixed kits, containing calcium silicate and phosphoserine (70% calcium silicate by molar percentage). Deionized water was added to start the adhesive reaction (0.5 cc per 2 grams of pre-mixed powders), and the adhesive was mixed manually for 30 s, before applying (0.4 cc) as a thick paste to the calcaneoquartal joint (Figure 1C). The joint was manually fixated for 90 s, then placed onto a flat surface to continue curing for 24 h at 21°C. The Model 1 group also included group with a weaker glue formulation (85% calcium silicate by molar percentage, Model 1-Weak), to serve as model validation if the present cadaver test model was sensitive enough to detect differences in glue strength.

In Model 3 the contributions of soft tissue to the failure strength, and to how the tissue dispersed load at and above the failure point of the glue, were evaluated by testing the joint first with glue (Model 3), then without glue (Model 3-without).

### 2.3. Mechanical testing

**Figure 2:**
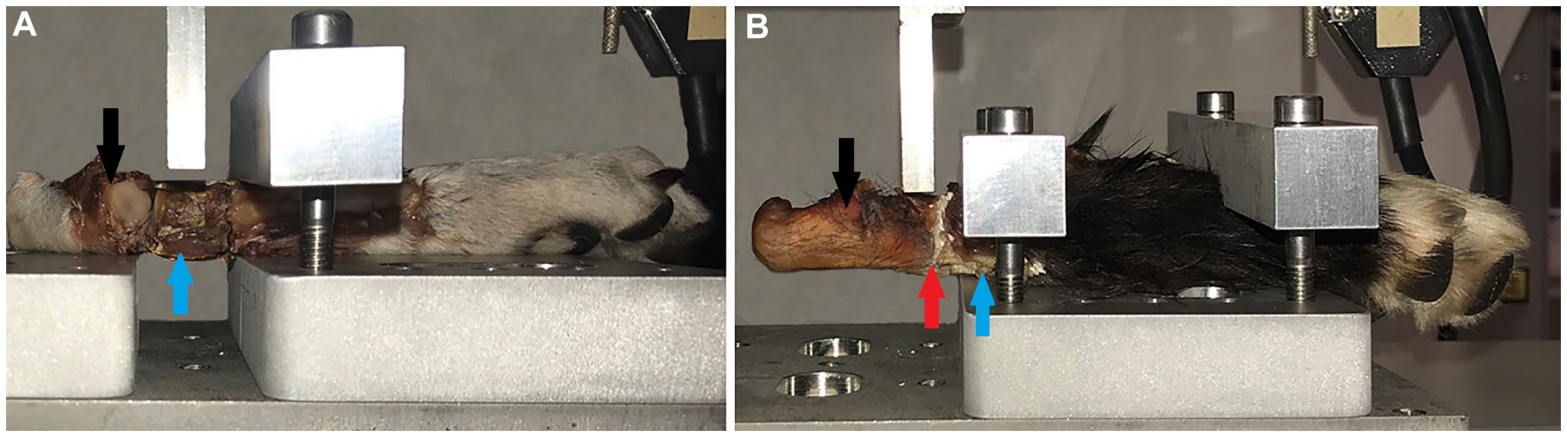
Mechanical testing rig and sample positioning. **(A)** The test rigg with a tarsal joint fixated in the test rig for shear testing. The impactor is positioned over the quartal bone (blue arrow), where the shear force was applied (Model 2–4). **(B)** Shows how tissues were prepared and tested for Model 1, with the metal impactor directly over calcaneus (black arrow) and the glued calcaneoquartal joint (red arrow). Backgrounds were edited using Photoshop, for increased visibility to the reader.

After curing for 24 h, the entire joint was loaded into a custom designed test rig, a 1 cm wide impactor was positioned over calcaneus (Model 1) or the quartal bone (Model 2–4), a shear force was applied via the impactor, and the failure strength was recorded from force-displacement curves (Figure 2). Data were obtained on an AGS-H, Shimadzu (Shimadzu Europa Gmbh, Duisburg, Germany) mechanical testing machine, using a displacement speed of 1 mm per minute, and a 5kN load cell. Data were analyzed using the manufacturer software, Trapezium-X Lite, version 1.2.0 (Shimadzu Europa).

To investigate if size of bonding surface affected performance of glue (i.e., differences in bond strength between dogs with differing joint size), all mechanical results were normalized to the average surface area of the two joint surfaces that were glued. Surface area was approximated as a square, by multiplying the diameter in the antero-posterior and medio-lateral directions and plotted in a scatter-plot analysis.

### 2.4. Statistics

The group means were compared using a one-way ANOVA, on JASP statistical software, version 0.16.0.0.

## 3. Results

In total, 35 tarsal joints where used (model 1 *n* = 9 and *n* = 8 “weak” glue, model 2 *n* = 8, model 3 *n* = 9, Model 4 *n* = 1).

In Model 1 the average adhesive strength, was 92.3 N/cm^2^, for the normal glue formulation (formulation: 70% calcium silicate by molar percentage). In Model 1-Weak with the weaker glue formulation (formulation: 85% calcium silicate by molar percentage), produced an average of 12.0 N/cm^2^ peak bond strength.

Using shear models that included more soft tissues, the average bond strength was slightly lower in both Model 2, at 79.7 N/cm^2^, and Model 3 with an average bond strength of 63.1 N/cm^2^. The difference in average bond strength, between models 1, 2, and 3 was not statistically significant (*p* = 0.37, ANOVA).

**Figure 3:**
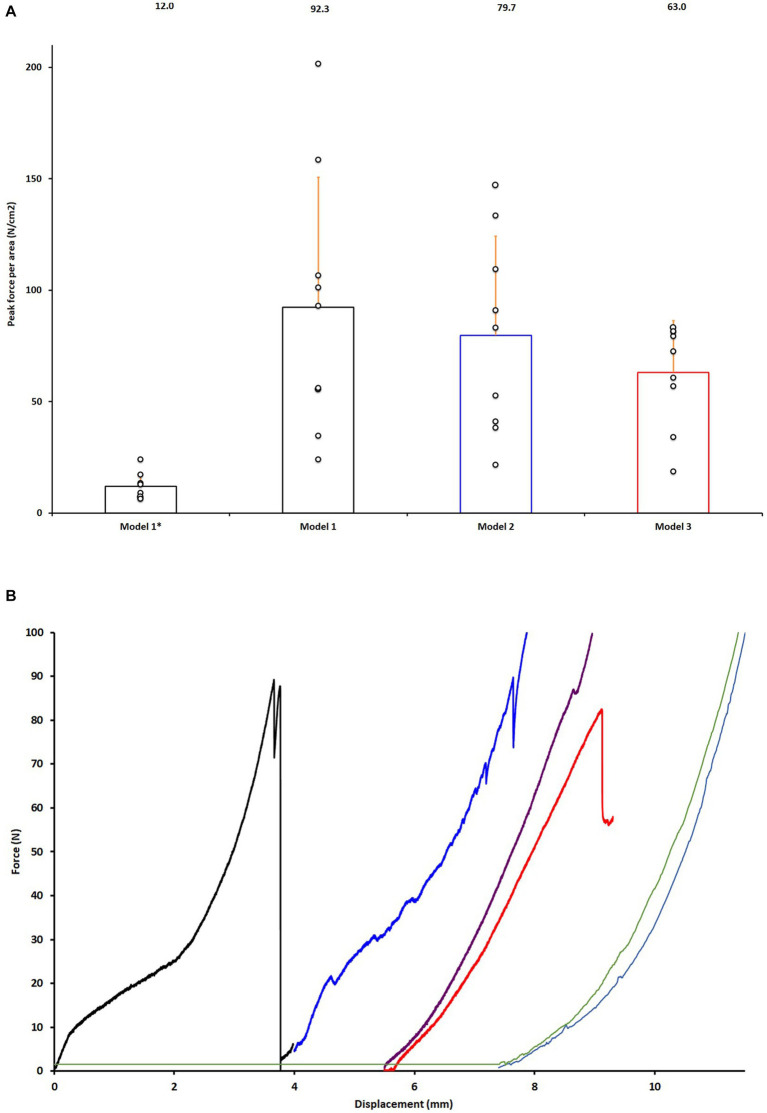
Validation and testing of models in glued joints. **(A)** Comparison of adhesive strength of the two glue formulations in Model 1-Weak (Model 1*) and Model 1 for validation of the test model and Model 2–3. Data is normalized to the average size of the two joint surface (force per square centimeter). **(B)** Representative force displacement curves of glued calcaneoquartal joints from data points shown in **(A)**, Model 3-without and Models 4. Black curve representing Model 1, blue curve Model 2, red curve Model 3, purple curve Model 3-without, light blue Model 4 and green Model 4-Glue.

The force displacement curves of Model 4/Model 4-Glue showed no point of failure, and there was no difference in force displacement curves (Figure 3).

**Figure 4:**
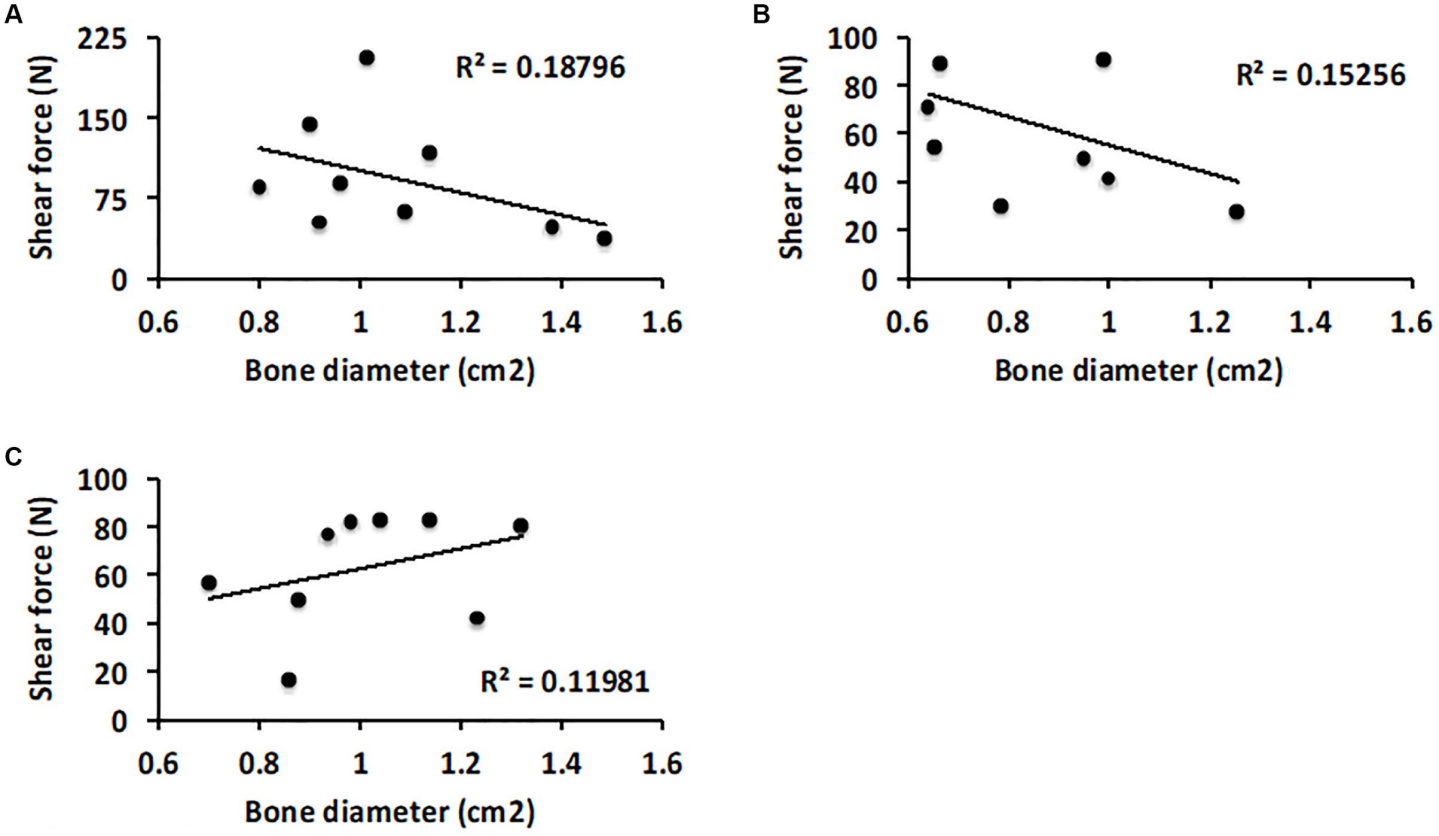
Scatter plot analysis of how the size of the tarsal joint surfaces correlate with performance of the glue. **(A)** Comparison of failure strength and average diameter of the calcaneus and quartal bone surfaces in Model 1. **(B)** Model 2 and **(C)** Model 3.

The average bone diameter (surface area) of calcaneus and quartal surfaces was 1.07 ± 0.23 (Model 1), 0.86 ± 0.21 (Model 2), and 1.01 ± 0.20 (Model 3) cm^2^. The correlation strength between the size of the animal (average bone diameter, surface area, of calcaneus and quartal surfaces was used as a proxy measure) and the bond strength of the glue, was poor (*R*^2^ < 0.20). While the average bond strength was slightly stronger for larger animals, when normalized by dividing the adhesive strength by the surface area, roughly equivalent bond strengths, per square centimeter, were obtained for both large and small animals (Figure 4).

## 4. Discussion

In this study we have developed a cadaveric biomechanical test model for calcaneoquartal arthrodesis sensitive enough to detect differences in glue strength and have shown that a new, phosphoserine modified, bone glue adds strength to the arthrodesis, *ex vivo*. This is the first study to evaluate a glue for fusing together subchondral bone surfaces. Our hypothesis that by adding the glue to an arthrodesis of the calcaneoquartal joint could add stability was confirmed.

Arthrodesis involves meticulous removal of articular cartilage, placement of a cancellous bone graft for its osteoconductive, osteoinductive, and osteogenic properties, and a rigid internal or external fixation to achieve osseous fusion (18, 19). Rigid fixation stability is the primary determinant of arthrodesis success, by promoting intramembranous ossification, while poor fixation leads to formation of fibrocartilage and non-union (20–25). In the human metatarsophalangeal joints, movement of the surfaces by 2 mm after arthrodesis is sufficient to interrupt healing, leading to non-union (26, 27). In the present study, joints treated with glue endured a displacement of 2–3 mm before the glue failed. In a clinical setting, the glue may be sufficient to reduce or prevent micro-movements between joint surfaces held together by orthopedic implants. A technology that reduces the risk of implant failures and non-union, especially in canine tarsal and carpal arthrodesis, where not all parts of the joint are fixated by the implant(s) could potentially be of great clinical value.

Another potential technique, not yet investigated, could be to fixate joints in correct alignment with the glue (solidifies in 60–90 s) before placing the implants during arthrodesis, thereby avoiding post malalignments, or misplacement of implants (6).

Historically the use of an external coaptation, usually a cast, to add rigidity and reduce the load of the internal implants has been advocated (8). The effectiveness of coaptation remains however unclear and recent studies suggest the contrary for tarsal and carpal arthrodesis (2, 28). A glue that adds stability to the fixation could even further diminish the need of an external coaptation, after it reached its full strength in 2–24 h depending on the formulation mix of the glue, in cases where it still is deemed necessary (29).

The glue evaluated in this study is a type of biomaterial: a ceramic glue that resorbs and is replaced by bone. The number of case reports evaluating biomaterials for arthrodesis are few and, to our knowledge, thus far there is no ceramic or polymeric biomaterial that can stabilize, fixate, or bond with subchondral bone (30–36). No resorbable material has achieved regulatory approval for use in gluing or fixating subchondral bone and there are no other case reports of an adhesive that can augment, or improve, arthrodesis procedures.

The developed cadaver model was sensitive enough to distinguish between glues with different strengths, and to identify failure of glue in the presence of some soft tissues, similar to the actual clinical conditions of arthrodesis. However, the presence of intact soft tissues contributed to stability and interfered with measurements in intact joints. We conclude, therefore, that biomechanical models of tarsal joint arthrodesis, which use an adhesive to bond or fixate joint surfaces, require removal of at least proximal soft tissues to identify failure of the glue and measure bond strength. The glue appeared to participate in load sharing with nearby soft tissues, similar to what would occur in fused joints *in vivo*.

After curing, the glue in this study resembles a ceramic material. Flaws or defects, such as pores, limit the structural strength of ceramics (37). The likelihood that defects arise during bonding or setting should increase with increases in the size of the bonding surface. Therefore, it was theoretically possible that the failure strength of the reconstructed joints might be correlated with the size of the joint. However, the bond strengths per square centimeter were similar for both small and large dogs.

The glue appeared strong enough to warrant further testing in joint arthrodesis, pending testing in a chronic (cyclical) loading model to account for premature failure due to fatigue. While it was only possible to evaluate the short term, immediate fixation strength (24 h) in the present model, in a recent study we have demonstrated that the adhesive bonds to live cancellous and cortical bone (*in vivo*) (14) just as strongly as to cadaver bone (13) in rodents and in human bone (38). Therefore, the results of this study are expected to closely predict how the glue will behave in live canines, within 24 h of fixation. However, in this study, rather than using tissue at physiological temperature (37°C), tissues were warmed to ambient temperature (21–22°C) to avoid variability. The use of warmed *ex vivo* tissues (37°C) would make the glue performance dependent on confounding variables affecting how quickly the tissues cools down, rather than on how the glue interacted with osteochondral tissue surfaces. Consequently, since the time for curing of the glue, not maximum bond strength, is affected by temperature with colder surfaces slowing the glue reaction, when used *in vivo* the adhesive will react faster and reach maximum strength sooner than in this study.

In laboratory testing, on metals and cortical bone, the average adhesive strength of the glue is typically 400 N/cm^2^ (15). This suggests that the joint fusion strength could be increased 2-4-fold (to reach the true strength of the glue) with additional improvements in the handling and fixation technique, or by optimizing the glue to bond with subchondral bone, rather than cortical bone, for future studies.

There were several limitations to the present model and data. First, the present cadaver model represented a compromise, between the need to remove all soft tissues and musculature to achieve a uniaxial loading regimen at a single bone/adhesive interface (e.g., pure shear). There was also a need to reproduce the clinical situation, where the injuries often are more complex, involving several joints and where the surrounding soft tissues and muscle disperse load, thereby preventing an accurate measurement of the biomechanical strength arising only from the bonded joint surfaces. *Ex-vivo* testing also did not consider changes in viscoelasticity between live tissue and cadaver tissue due to decomposition (cadaver), temperature, healing, inflammation, or the presence of fluids that can affect the material properties in live tissue.

Another limitation in the present study was the type of loading forces. A purely shear force was used because this is the most rigorous test for a glue (i.e., most glues are weakest in shear), in addition to being a force that arises during normal locomotion, besides compression, bending and torsion. While tarsal loading has been evaluated in general by using: 1-, 2-, and 3-point bending (26, 39–41); pure torsion (24); compression (42); shear (27) and multi-modal loading using intact (cadaver) ankles (43) there is no clear consensus on which type(s) of loading are sufficient to accurately model forces that arise during locomotion. No other studies have evaluated adhesives for joint fixation, and the magnitude of force(s) that arise during joint movement are poorly characterized in canines. The most comprehensive study thus far in humans, Wang et al. (25) found that the expected peak load in human calcaneocuboidal joints during movement was equivalent to 9% of bodyweight (0.3–0.5 MPa or 40-100 N). Directly translating the results from Wang et al. (25) from human to canine, is impossible due to differences in movement patterns, anatomy, and functional loading angles, between bipeds and quadrupeds. However, a reasonable assumption of loading in canine calcaneoquartal joint is that it would not be greater than in humans. Based upon this assumption the expected peak loading force in a toy breed (weighing 1–3 kg) might be 1–3 N, while a medium sized dog (5–20 kg) might exert up to 20 N on the calcaneoquartal joint during movement. This range of loading forces would be much lower than the measured strength of the glue. Both the joint and surrounding soft tissues disperse load, which may reduce the forces acting upon the joint surfaces, while also complicating analysis of precisely how strong a glue must be to resist displacement/ deformation, and to ensure joint fusion.

Finally, since we evaluated a joint where the surfaces were not visible during testing, failure of the glued joint was confirmed visually when cracks appeared in the bond line. It is possible that smaller, or partial, failures could have occurred in the glue (e.g., fatigue), when it was subjected to repeated loading. Additional studies are needed to evaluate the fatigue properties of the glue. While not obvious from the data or discussion thus far, the largest challenge in this study was developing a manual fixation method to hold the uneven joint surfaces steady, and avoid disrupting the adhesive while it cured, during the 60–90 s curing/working time. The joint surfaces were not flat, nor perfectly aligned, which is a crucial impediment for any glue, which make the results of this study even more relevant.

## 5. Conclusion

A cadaver model, of canine calcaneoquartal joint arthrodesis was sensitive enough to measure immediate fixation strength of bone adhesive-type materials. We have also shown that the bone glue produces strong fixation in cadaver tissue with minimal manual fixation time. Finally, the adhesive strength could be sufficient for *in vivo* short-term loading by small and medium breeds (20 kg), supporting future evaluation in live animals.

## Author’s note

An abstract was presented at the 2022 ACVS Surgery Summit, October 13–15, Portland, Oregon, USA. https://doi.org/10.1111/vsu.13879.

## Data availability statement

The original contributions presented in the study are included in the article/supplementary material, further inquiries can be directed to the corresponding author.

## Ethics statement

Ethical approval for use of cadavers was obtained from the Uppsala Animal Ethics Committee, 15533-2018, 04682-2020. The cadavers were donated by owners via a signed consent form (at the time for the dog’s euthanasia).

## Author contributions

TL prepared the specimen, interpreted data and drafted, revised, edited, and completed the manuscript. MP-P prepared specimen, performed and analyzed experiments of mechanical properties, drafted, revised, and edited the manuscript. GS prepared specimen, interpreted data, drafted, revised, and edited the manuscript. OH collected specimen, interpreted data, drafted, revised, and edited the manuscript. All authors contributed to the article and approved the submitted version.

## References

[1] Abrams BE Wavreille VA Hettlich BF Selmic LE. Corrective osteotomy and partial tarsal arthrodesis in two greyhounds with calcaneal malunion. Vet Surg. (2020) 49:1600–8. doi: 10.1111/vsu.13517, PMID: 33009862

[2] Anesi S Clarke S Gemmill T Oxley B Pink J Smith K. Long-term outcomes after pantarsal arthrodesis with medial plate fixation without external coaptation in 30 dogs. Vet Surg. (2020) 49:502–11. doi: 10.1111/vsu.13354 31769056

[3] Franch J Lafuente P Diaz-Bertrana MC Munilla A Durall I Pastor J. Management of leishmanial osteolytic lesions in a hypothyroid dog by partial tarsal arthrodesis. Vet Rec. (2004) 155:559–62. doi: 10.1136/vr.155.18.559 15559422

[4] Massimo P Michela B. Pantarsal arthrodesis to treat a full-thickness lateral trochlear ridge osteochondritis dissecans in a dog. VCOT Open. (2019) 2:e27–31. doi: 10.1055/s-0039-1687885

[5] Ost PC Dee JF Dee LG Hohn RB. Fractures of the calcaneus in racing greyhounds. Vet Surg. (1987) 16:53–9. doi: 10.1111/j.1532-950X.1987.tb00913.x 3507125

[6] Roch SP Clements DN Mitchell RAS Downes C Gemmill TJ Macias C. Complications following tarsal arthrodesis using bone plate fixation in dogs. J Small Anim Pract. (2008) 49:117–26. doi: 10.1111/j.1748-5827.2007.00468.x, PMID: 18086158

[7] Barnes DC Knudsen CS Gosling M McKee M Whitelock RG Arthurs GI. Complications of lateral plate fixation compared with tension band wiring and pin or lag screw fixation for calcaneoquartal arthrodesis. Treatment of proximal intertarsal subluxation occurring secondary to non-traumatic plantar tarsal ligament disruption in dogs. Vet Comp Orthop Traumatol. (2013) 26:445–52. doi: 10.3415/VCOT-12-07-0089, PMID: 24008374

[8] Klause SE Piermattei DL Schwarz PD. Tarso-crural arthrodesis: complications and recommendations. Vet Comp Orthop Traumatol. (1989) 2:119–24. doi: 10.1055/s-0038-1633208

[9] Choi CJ Balara JM Casale SA Wendelburg KL. Implant removal rate after partial carpal arthrodesis in dogs: a retrospective analysis of 22 cases. Front Vet Sci. (2023) 10:10. doi: 10.3389/fvets.2023.1160129, PMID: 37082137 PMC10110909

[10] Bristow WR. Arthrodesis. Brit J Surg. (1928) 15:401–13. doi: 10.1002/bjs.1800155908

[11] McKee WM May C Macias C Lapish JP. Pantarsal arthrodesis with a customised medial or lateral bone plate in 13 dogs. Vet Rec. (2004) 154:165–70. doi: 10.1136/vr.154.6.165, PMID: 14979670

[12] Hulsart-Billström G Stelzl C Procter P Pujari-Palmer M Insley G Engqvist H. *In vivo* safety assessment of a bio-inspired bone adhesive. J Mater Sci-Mater M. (2020) 31:24. doi: 10.1007/s10856-020-6362-3, PMID: 32036502 PMC7007900

[13] Procter P Pujari-Palmer M Hulsart-Billström G Wenner D Insley G Larsson S. A biomechanical test model for evaluating osseous and osteochondral tissue adhesives. BMC Biomed Eng. (2019) 1:11. doi: 10.1186/s42490-019-0011-2, PMID: 32903290 PMC7422571

[14] Procter PH-BG Alves A Pujari-Palmer M Wenner D Insley G Engqvist H. Gluing living bone using a biomimetic bioadhesive: from initial cut to final healing. Front Bioeng Biotechnol. (2021) 9:9. doi: 10.3389/fbioe.2021.728042, PMID: 34820360 PMC8606677

[15] Pujari-Palmer M Giró R Procter P Bojan A Insley G Engqvist H. Factors that determine the adhesive strength in a bioinspired bone tissue adhesive. Chem Eng. (2020) 4:1–19. doi: 10.3390/chemengineering4010019

[16] Pujari-Palmer M Guo H Wenner D Autefage H Spicer CD Stevens MM. A novel class of injectable bioceramics that glue tissues and biomaterials. Materials. (2018) 11:1–15. doi: 10.3390/ma11122492, PMID: 30544596 PMC6316977

[17] Evans HE de Lahunta A. Miller’s anatomy of the dog. 4th ed. St Louis: Saunders/Elsevier (2013).

[18] Ogawa T Ishii T Mishima H Sakai S Watanabe A Nishino T. Effectiveness of bone marrow transplantation for revitalizing a severely necrotic small bone: experimental rabbit model. J Orthop Sci. (2010) 15:381–8. doi: 10.1007/s00776-010-1459-z 20559807

[19] Johnson KA Bellenger CR. Effects of autologous bone grafting on bone healing after carpal arthrodesis in the dog. Vet Rec. (1980) 107:126–32. doi: 10.1136/vr.107.6.126, PMID: 7003910

[20] Bennett GL Cameron B Njus G Saunders M Kay DB. Tibiotalocalcaneal arthrodesis: a biomechanical assessment of stability. Foot Ankle Int. (2005) 26:530–6. doi: 10.1177/107110070502600706, PMID: 16045843

[21] Buda M Hagemeijer NC Kink S Johnson AH Guss D DiGiovanni CW. Effect of fixation type and bone graft on tarsometatarsal fusion. Foot Ankle Int. (2018) 39:1394–402. doi: 10.1177/1071100718793567, PMID: 30175622

[22] Dock CC Freeman KL Coetzee JC McGaver RS Giveans MR. Outcomes of nitinol compression staples in tarsometatarsal fusion. Foot Ankle Ortho. (2020) 5:1–6. doi: 10.1177/2473011420944904, PMID: 35097401 PMC8697117

[23] Lareau CR Deren ME Fantry A Donahue RMJ DiGiovanni CW. Does autogenous bone graft work? A logistic regression analysis of data from 159 papers in the foot and ankle literature. Foot Ankle Surg. (2015) 21:150–9. doi: 10.1016/j.fas.2015.03.008 26235852

[24] Riedl M Glisson RR Matsumoto T Hofstaetter SG Easley ME. Torsional stiffness after subtalar arthrodesis using second generation headless compression screws: biomechanical comparison of 2-screw and 3-screw fixation. Clin Biomech. (2017) 45:32–7. doi: 10.1016/j.clinbiomech.2017.04.004, PMID: 28458187

[25] Wang Y Li Z Wong DW-C Zhang M. Effects of ankle arthrodesis on biomechanical performance of the entire foot. PLoS One. (2015) 10:1–22. doi: 10.1371/journal.pone.0134340, PMID: 26222188 PMC4519327

[26] Campbell B Schimoler P Belagaje S Miller MC Conti SF. Weight-bearing recommendations after first metatarsophalangeal joint arthrodesis fixation: a biomechanical comparison. J Orthop Surg Res. (2017) 12:23. doi: 10.1186/s13018-017-0525-z, PMID: 28166805 PMC5294903

[27] Milshteyn MA Dwyer M Andrecovich C Bir C Needleman RL. Comparison of two fixation methods for arthrodesis of the calcaneocuboid joint: a biomechanical study. Foot Ankle Int. (2014) 36:98–102. doi: 10.1177/1071100714552479, PMID: 25384391

[28] Bristow PC Meeson RL Thorne RM Butterworth SJ Rutherford S Renwick AIC. Clinical comparison of the hybrid dynamic compression plate and the Castless plate for Pancarpal arthrodesis in 219 dogs. Vet Surg. (2015) 44:70–7. doi: 10.1111/j.1532-950X.2014.12183.x 24708556

[29] Meeson RL Davidson C Arthurs GI. Soft-tissue injuries associated with cast application for distal limb orthopaedic conditions. Vet Comp Orthop Traumatol. (2011) 24:126–31. doi: 10.3415/VCOT-10-03-0033, PMID: 21225085

[30] Beuchel MW Brage M. Bone reconstitution with synthetic bone graft material for osseous defects in revision total ankle arthroplasty. Foot & Ankle Orthopaedics. (2016) 1. doi: 10.1177/2473011416S00139

[31] Bitterman A Mathew C Patel M Gurtowski JP. Antibiotic spacer arthroplasty for revision MTP arthrodesis: a novel means to build the implant: a case report. Cureus. (2016) 8:e537. doi: 10.7759/cureus.537, PMID: 27114892 PMC4841614

[32] Gonçalves H Pascal-Moussellard H Lesoeur J Schnitzler V Fellah BH Wagner NMS. Injection of calcium phosphate apatitic cement/blood composites in intervertebral fusion cages: a simple and efficient alternative to autograft leading to enhanced spine fusion. Spine. (2020) 45:E1288–95. doi: 10.1097/BRS.0000000000003598, PMID: 32694485

[33] Klos K Wähnert D Gueorguiev B Schwieger K Hofmann GO Windolf M. Development of a technique for cement augmentation of nailed tibiotalocalcaneal arthrodesis constructs. Clin Biomech. (2010) 25:576–81. doi: 10.1016/j.clinbiomech.2010.03.006, PMID: 20385434

[34] Liverneaux P Khallouk R. Calcium phosphate cement in wrist arthrodesis: three cases. J Orthop Sci. (2006) 11:289–93. doi: 10.1007/s00776-006-1008-y 16721532

[35] O’Donnell SW Vaughn JJ Sangeorzan AP DeFontes KW III Bluman EM. Surgical strategies: cement spacer for staged first metatarsophalangeal arthrodesis after failed arthroplasty. Tech Foot Ankle Surg. (2020) 19:225–9. doi: 10.1097/BTF.0000000000000252

[36] Pomeroy G De Ben S. Ankle arthrodesis with silicate-substituted calcium phosphate bone graft. Foot Ankle Online J. (2013) 6:2. doi: 10.3827/faoj.2013.0601.002

[37] Pujari-Palmer M Robo C Persson C Procter P Engqvist H. Influence of cement compressive strength and porosity on augmentation performance in a model of orthopedic screw pull-out. J Mech Behav Biomed. (2018) 77:624–33. doi: 10.1016/j.jmbbm.2017.10.016, PMID: 29100205

[38] Bojan AJ Stadelmann VA Wu D Pujari-Palmer M Insley G Sundh D. A new bone adhesive candidate- does it work in human bone? An *ex-vivo* preclinical evaluation in fresh human osteoporotic femoral head bone. Injury. (2022) 53:1858–66. doi: 10.1016/j.injury.2022.04.007, PMID: 35469636

[39] Aiyer A Russell NA Pelletier MH Myerson M Walsh WR. The impact of nitinol staples on the compressive forces, contact area, and mechanical properties in comparison to a claw plate and crossed screws for the first tarsometatarsal arthrodesis. Foot Ankle Spec. (2015) 9:232–40. doi: 10.1177/1938640015620655, PMID: 26655080

[40] Rodriguez R Dun S He JK McKissack H Fleisig GS Jain M. Biomechanical comparison of plantar-to-dorsal and dorsal-to-plantar screw fixation strength for subtalar arthrodesis. Foot & Ankle Orthopaedics. (2019) 4. doi: 10.1177/2473011419S00363 PMC7046341 32159606

[41] Wininger FA Kapatkin AS Radin A Shofer FS Smith GK. Failure mode and bending moment of canine pancarpal arthrodesis constructs stabilized with two different implant systems. Vet Surg. (2007) 36:724–8. doi: 10.1111/j.1532-950X.2007.00326.x, PMID: 18067612

[42] Jastifer JR Alrafeek S Howard P Gustafson PA Coughlin MJ. Biomechanical evaluation of strength and stiffness of subtalar joint arthrodesis screw constructs. Foot Ankle Int. (2015) 37:419–26. doi: 10.1177/1071100715619680, PMID: 26635413

[43] Simons P Sommerer T Zderic I Wahl D Lenz M Skulev H. Biomechanical investigation of two plating systems for medial column fusion in foot. PLoS One. (2017) 12:e0172563. doi: 10.1371/journal.pone.0172563, PMID: 28222170 PMC5319781

